# Evolution During Bottle Ageing of Wines Macerated with Toasted Vine-Shoots and Micro-Oxygenation

**DOI:** 10.3390/biom14111372

**Published:** 2024-10-28

**Authors:** Cristina Cebrián-Tarancón, Rosario Sánchez-Gómez, Ana María Martínez-Gil, Maria del Álamo-Sanza, Ignacio Nevares, Maria Rosario Salinas

**Affiliations:** 1 Cátedra de Química Agrícola, E.T.S. de Ingeniería Agronómica y de Montes y Biotecnología, Universidad de Castilla-La Mancha, Avda. de España s/n, 02071 Albacete, Spain; cristina.ctarancon@uclm.es (C.C.-T.); rosario.salinas@uclm.es (M.R.S.); 2 Departamento de Química Analítica, UVaMOX—Universidad de Valladolid, 34004 Palencia, Spain; anamaria.martinez.gil@uva.es (A.M.M.-G.); maria.alamo.sanza@uva.es (M.d.Á.-S.); 3 Departamento de Ingeniería Agroforestal, UVaMOX—Universidad de Valladolid, 34004 Palencia, Spain; ignacio.nevares@uva.es

**Keywords:** bottling time, SEGs-MOX combination, phenolic and volatile compounds, oxygen transmission rate

## Abstract

The effects of SEGs (“Shoot from vines—Enological—Granule”) on winemaking within the same variety are well established. However, the interaction of different SEG varieties combined with micro-oxygenation (MOX) and its subsequent evolution in the bottle has not been investigated to date. In this work, Tempranillo wines were treated with two doses of SEGs from Tempranillo and Cabernet Sauvignon (12 and 24 g/L) and subjected to two fixed MOX doses (LOTR, 6.24 mg/L·month, and HOTR, 11.91 mg/L·month). After that, the wines were bottled, and their chemical composition and sensory profile were analysed after 3 and 6 months. Although no clear trend directly associated with the use of MOX was observed, in terms of chemical composition, wines showed an evolution in their chemical profile over time, with compounds such as vanillin increasing as more oxygen was added. Regarding their sensorial profile, the wines were more rounded after 6 months that in bottling, where SEGs or toasted descriptors, studied at the taste phase, were slightly more intense with the low SEG dose and HOTR combination.

## 1. Introduction

The wine sector has been undergoing a great transformation in recent decades, due to the research and technological progress whose objective has been to integrate sustainability with enhancements in the quality and differentiation of wines. Hence, the continuous priority for winemakers and researchers worldwide is the search for original approaches and methodologies to enhance the sensorial attributes of wines.

Regarding these innovate techniques, the use of toasted vine-shoots as oenological additives (known as SEGs) represents a revolution in the sector. The oenological potential of this wood has been extensively proven thanks to numerous factors studied, among which are aspects related to the wood itself, such as fragment size and toasting level, as well as others related to the winemaking process, such as dosage, contact time, timing of addition, wine variety, and the combination with different extraction techniques [1,2,3,4,5,6]. In all cases, the results indicate a significant exchange of phenolic and volatile compounds from the SEGs to the wines, also promoting tannin evolution, and, therefore, influencing the chemical and sensorial profile of the final wines. Moreover, some studies have also been carried out on the evolution of these wines in the bottle, showing a greater integration of the wood SEG compounds, as well as a softer and more pleasant sensorial profile for the tasters, setting 6 months as the optimum time in the bottle ageing [7,8].

All this research has been developed using vine-shoots from the same variety as the wine to which they were added; for example, Tempranillo vine-shoots to wines made from Tempranillo grapes variety. However, the specific impact of toasted vine-shoots from different varieties on wine remains unstudied currently. However, as with the different grape varieties, it is known that the varietal character is also manifested in the phenolic and volatile composition of the vine-shoots [9,10]. So, diversifying the SEGs used can provide a wide range of phenolic and aroma compounds different from the varietals of the wine used, which would broaden the possibility to innovate, create unique profiles, and respond to changing market demands, thus enriching the consumer’s sensorial experience.

Conversely, micro-oxygenation (MOX) is an innovative oenological technique with a significant potential for influencing the structure, aroma profile, and stability of wines [11,12,13,14]. This method entails the controlled and precise introduction of small quantities of oxygen during the ageing process and is primarily employed alongside alternative oak products [15,16,17]. Oxygen plays a crucial role in facilitating reactions like those that occur within barrels during the ageing of wine. So, in case of wines elaborated in contact with toasted vine-shoots fragments, the implementation of this technique could affect the overall quality and characteristics of the final wine. However, the first studies of wines in contact with SEGs and MOX did not show a clear influence at bottling time [18]. This may be due to the fact that, after wine maceration with wood and MOX, a bottle period is necessary for wines which undergo changes that began during wood contact, which are largely determined by the wood, their pool of potential extractable compounds, and the contact period [19,20,21]. However, this evolution in the bottle has not yet been studied in SEGs-MOX wines.

From the sensorial point of view, bottle ageing improves the wine characteristics since wines acquire a more delicate aroma, due to new flavour compounds which may be formed, while the concentrations of others, originally present, may increase or decrease. Also, a higher roundness and harmony in the taste perceived, decreasing the intensity of the woody character and astringency. From the chemical point of view, bottle ageing involves numerous reactions, which may drastically affect the wines’ characteristics, such as oxidation, esterification, and hydrolysis reactions, among others. This leads to bottle ageing being an important process affecting the flavour quality development of wine maturation, highlighting a first stage that involves wine maturation, wherein wine taste and aroma are improved, followed by an evolution stage that is when the wine has the best sensorial quality [22].

Consequently, this research examines how the combination of SEGs from Tempranillo and Cabernet Sauvignon varieties with MOX treatments affects the phenolic and volatile composition, along with the sensory characteristics of Tempranillo wines during the ageing process in bottle. For that, different SEGs and MOX treatments were developed after vinification and wines were analysed after 3 and 6 months in the bottle.

## 2. Materials and Methods

Since this work is the continuation of the authors’ research, for which previous data can be found in Cebrián-Tarancón et al., 2023 [18], the same experiments conditions were used for wine, SEGs and MOX treatments and wine analysis.

### 2.1. Wine

Tempranillo grapes, which were harvested at their optimal maturation moment, underwent destemming and crushing procedures. Subsequently, the resulting musts were concurrently inoculated with the commercially available *Saccharomyces cerevisiae* strain Uvaferm HPS active dry yeast (Lallemand, St Simon, France) for the initiation of alcoholic fermentation, and the commercial *Oenococcus oeni* strain Lalvin VP41 (Lallemand, St Simon, France) to facilitate malolactic fermentation. Vinification was carried out under controlled temperature (18 °C for alcoholic fermentation and 21 °C for malolactic fermentation) employing stainless-steel tanks, with two pump-ups by day, each lasting one minute. The alcoholic fermentation process was monitored over a duration of 10 days by daily assessments of must temperature and density. Malic acid levels were quantified every 2 days, and once concentrations fell below 0.1 g/L, malolactic fermentation was deemed complete, after 10 days from commencement. Following malolactic fermentation, potassium metabisulfite was added after malolactic fermentation to give a total SO_2_ concentration of 75 mg/L.

### 2.2. SEGs and MOX Treatments

Vine-shoots were collected from two red *Vitis vinifera* varieties at the Pago de La Jaraba winery in Castilla-La Mancha, Spain: Tempranillo (T; VIVC: 12350) and Cabernet Sauvignon (CS; VIVC: 1929). Following the pruning process, the vine-shoots were kept whole in a dark environment at room temperature (18 ± 3 °C) for six months. To convert vine-shoots into an oenological additive, they were processed into granules and referred to as SEGs (Shoots from vines—Enological—Granule). Wood underwent grinding using a hammer miller (Skid Sinte 1000; LARUS Impianti, Zamora, Spain), resulting in particle sizes ranging from 2 mm to 2 cm. Subsequently, the ground material was subjected a toasting process in an oven equipped with air circulation (Heraeus T6; Heraeus, Hanau, Germany) at 180 °C for 45 min, following the method outlined by Cebrián-Tarancón et al. [4]).

For the combined treatments utilising SEGs and micro-oxygenation (MOX) [18], 20-L stainless steel tanks were employed, each with a polydimethylsiloxane (hereinafter, PDMS) infusion tube (RedOaker, West Lakes Shore, South Australia, Australia) of various lengths to ensure an adequate oxygen transfer rate (OTR). Two OTR levels were defined: low (L), with 6 mg/L·month, and high (H), with 12 mg/L·month. To activate this MOX system, PDMS infusion tubes were linked to a (Barrel)mate device (Wine Network Technology Pty Ltd., South Australia, Australia), achieving an application of 6.24 ± 0.87 mg/L⋅month in LOTR and 11.91 ± 0.71 mg/L⋅month in HOTR.

SEGs were introduced in infusion bags and subsequently into the wines (which had already completed malolactic fermentation) at two different doses (12 and 24 g/L) and were in contact with wines and MOX supply for 35 days according to Cebrián-Tarancón et al. [2]. Once vine-shoots were removed, wines were bottled and analysed at this time (B) (data already published, [18]) and after 3 (3 M) and 6 months (6 M) of bottle ageing. All test conditions were carried out in triplicate.

This study was conducted with the following combinations (SEGs variety, SEGs dose, and MOX dosage), as shown in Table 1:

### 2.3. Wine Analysis

#### 2.3.1. General Oenological Parameters

Oenological parameters such as alcohol concentration (% *v*/*v*), titratable acidity (TA, expressed as g/L of tartaric acid), pH, volatile acidity (VA, expressed as g/L of acetic acid), reducing sugar (RS, expressed as g/L), and total SO_2_ (T–SO_2_, expressed as mg/L) were measured in all wines previous to bottling and after 3 and 6 months of bottle ageing, following the methods established by the International Organisation of Vine and Wine [23].

#### 2.3.2. Determination of Volatile Compounds by SBSE-GC-MS

Wine volatiles were determined according to the methodology of Sánchez-Gómez et al. [10]. The extraction of these compounds was carried out using Stir Bar Sorptive Extraction of PDMS coating (10 mm length; 0.5 mm film thickness). Wines were stirred at 500 rpm for 60 min at room temperature (21 ± 3 °C). After the extraction period, Stir Bars were washed with distilled water and dried with a tissue. Analysis was performed using an automated thermal desorption unit (TDU, Gerstel, Mülheim and der Ruhr, Germany) mounted on an Agilent 7890A gas chromatograph system (GC) coupled to a quadrupole Agilent 5975C electron ionisation mass spectrometric detector (MS, Agilent Technologies, Palo Alto, CA, USA). The GC system was equipped with a fused silica capillary column (BP21 stationary phase; 30 m length; 0.25 mm I.D.; and 0.25 μm film thickness) sourced from SGE (Ringwood, Australia). Helium was employed as the carrier gas, maintaining a constant column pressure of 20.75 psi.

The Stir Bars were thermally desorbed within a helium carrier gas, flowing a rate of 75 mL/min with the TDU programmed to increase from 40 to 295 °C (maintained for 5 min) at a ramp rate of 60 °C/min at splitless desorption mode. The analytes were concentrated within a programmed temperature vaporising injector (PTV) (CIS-4, Gerstel), incorporating a packed liner (20 mg tenax TA), held at −10 °C with cryo cooling prior to injection. Following desorption and concentration, the CIS-4 was programmed from −10 °C to 260 °C (held for 5 min) at a ramp rate of 12 °C/min to transfer the captured volatiles onto the analytical column. The GC oven temperature was programmed to 40 °C (held for 2 min), gradually increased to 80 °C (5 °C/min, held for 2 min), further elevated to 130 °C (10 °C/min, held for 5 min), then to 150 °C (5 °C/min, held for 5 min), and finally to 230 °C (10 °C/min, held for 5 min). MS analysis was performed using scan acquisition (*m*/*z* 27–300) with an ionisation energy of 70 eV. The temperature of the MS transfer line was maintained at 230 °C. MS data acquisition was operated in positive scan mode to avoid matrix interferences, and the MS quantification was executed in single ion-monitoring mode using their characteristic *m*/*z* values of each compound. Detailed information regarding to the analysed compounds and their corresponding *m*/*z* vales can be found in work of Sánchez-Gómez et al. [10]. Compounds identification was achieved using the NIST library and subsequently confirmed comparting the mass spectra and retention time with those of pure standards for each compound (Sigma-Aldrich, Steinheim, Germany). The internal standard used was 3-methyl-1-pentanol. Quantification was based on calibration curves elaborated with five different concentrations of the corresponding standards (R^2^ = 0.95–0.97). All analyses were performed in triplicate.

#### 2.3.3. Determination of Low Molecular Weight Phenolic Compounds by HPLC-DAD

Low molecular weight phenolic compounds of wines were conducted following the work by Cebrián-Tarancón et al. [1]. First, samples were subjected to filtration through a 0.22 µm PVDF filter, after which a volume of 20 μL of wine was injected into an Agilent 1200 HPLC chromatograph (Palo Alto, CA, USA) equipped with a Diode Array Detector (DAD, Agilent G1315D, Palo Alto, CA, USA) connected to an Agilent ChemStation (version B.03.01) data-processing station. Separation was performed using a reverse-phase ACE C18-PFP (4.6 mm × 150 mm, 3 μm particle size) coupled with a ACE Excel HPLC pre-column Filter 1PK (0.5 μm particle size), maintained at 30 °C.

The HPLC elution solvents included a mixture of water/formic acid/acetonitrile (97.5:1.5:1 *v*/*v*/*v*) as solvent A and acetonitrile/formic acid/solvent A (78.5:1.5:20 *v*/*v*/*v*) as solvent B. The elution gradient for solvent B was programmed as follows: 0 min, 5%; 8.40 min, 5%; 12.50 min, 10%; 19 min, 15%; 29 min, 16%; 30 min, 16.5%; 34.80 min, 18%; 37.20 min, 32%; 42 min, 62%; 52 min, 90%; 54 min, 100%; 56 min, 100%; 60 min, 5%; 65 min, 5%.

Detection of all compounds was carried out using the DAD detector, comparing the UV–Vis spectra and retention times of the compounds with those of their corresponding pure standards (Sigma-Aldrich, Steinheim, Germany). Detailed information regarding the quantification wavelength of the analysed compounds can be found in the work of Cebrián-Tarancón et al. [1]. Quantification was based on the calibration curves elaborated from five different concentrations of the respective standards, determined by UV–Vis signal (R^2^ = 0.92–0.99). All analyses were conducted in duplicate.

#### 2.3.4. Sensory Analysis of the Wines

A panel of sixteen wine experts, consisting of seven females and nine males aged between 25 and 65 years old, participated in the tasting. Prior to the tasting sessions, a training procedure was conducted according to the work by Sanchez-Gómez et al. [8]. Sensory analyses were carried out at three distinct time points: pre-bottling and after 3 and 6 months of bottle ageing. During the tasting, the eight wines from the treatments were evaluated and compared to the initial control wine (bottling time).

Each tasting session had two rounds: first, wines from Tempranillo SEGs (TE12-LOTR, TE12-HOTR, TE24-LOTR, TE24-HOTR), and second, wines from Cabernet Sauvignon SEGs (CS12-LOTR, CS12-HOTR, CS24-LOTR, CS24-HOTR). The sequence of wines sampling, unknown to the experts, started from the lowest to highest SEGs dose. Within each dose, wines were tasted from less (LOTR) to more micro-oxygenation (HOTR). The environment was kept at 21 °C with air conditioning, and tasters were in isolated booths. To assess treatment effects, three bottles from the same treatments were combined before each session, ensuring uniformity, and discarding any that differed. Thus, 9 wines were tested at each tasting time. An adapted evaluation sheet, developed by consensus in a specific session, was used including new descriptors to characterise SEGs aroma. Judges assessed each wine using 8 descriptors grouped by olfactory phase (red fruits, nuts, vanilla, toasted, and SEGs), taste phase (red fruits, nuts, vanilla, toasted, and SEGs), and tannins (dryness, silkiness, and bitterness).

A volume of 30 mL from each wine sample was assessed in winetaster glasses at a temperature of 21 °C in accordance with the standard ISO 3591 (1977) [24]. Wine experts were supplied with unsalted crackers and water, and they were instructed to rinse their mouths and wait for at least two minutes between tasting each sample. After that, the experts smelled and tasted the different wines, recorded the specific descriptors perceived, and rated the intensity of each sensorial descriptor on an 11-point scale. In this scale, a score of 0 indicated that the descriptor was not detected (absence), while scores ranging from 1 to 10 represented intensity from very low to maximum, respectively. All sensory evaluations were carried out under Spanish Standardisation Rules (6658:2017, 1997) [25].

### 2.4. Data Analysis

Statistical analyses were performed with Statgraphics Centurion statistical program (version 19.4.02; StatPoint, Inc., The Plains, VA, USA). First, a principal component analysis (PCA) was performed with the purpose of obtaining an overall view of the influence of SEGs-MOX treatments in wines and the evolution of these after 6 months in bottle. Moreover, a comparison between the general parameters of all wines was carried out and, on the other hand, a more detailed analysis was performed for each wine separately. On the latter, for each wine, the differences between the two doses of MOX (LOTR and HOTR) were compared with respect to the bottling time. In addition, for each fixed dose of MOX, the two doses of SEGs (12 and 24 g/L) were compared to evaluate their effect. In both cases, a one-way analysis of variance (ANOVA) was conducted at a 95% probability level, according to Fisher’s least significant difference (LSD) test.

## 3. Results and Discussion

### 3.1. Oenological Parameters

Although in oenology terms the differences between general parameters may not be noticeable for practical purposes, statistically significant differences between wines after 3 and 6 months of bottle ageing were observed based on the SEGs-MOX treatments (Table 2). ANOVA was performed comparing the parameters values of all wines for each sampling time (3 and 6 months). Concerning the variation in the alcohol concentration (% *v*/*v*), the higher values were found in wines with the lower dose of Tempranillo SEGs. Slightly higher total acidity values were observed for CS12-HOTR wine, both at 3 and 6 months in bottle, and in the TE12-LOTR wine in the latter sampling period. Regarding pH, wines elaborated with the highest SEGs-MOX doses combination (24 g/L and HOTR) showed the highest values after 3 months in bottle, but such differences were reduced after 6 months in bottle (Table 2). As regards volatile acidity, at 3 months in bottle, the highest value was observed with the Cabernet Sauvignon SEGs (CS12-HOTR). However, after 6 months of bottling, the values of wines with the highest SEGs-MOX doses combination (TE24-HOTR and CS24-HOTR) increased to the same level as the previous one. Reducing sugar showed a higher increase when a higher SEGs dose was used, since vine-shoots constitute an abundant lignocellulosic source [26]. Although those values were quite close to 2 g/L or even higher, it would not be considered a problem for a possible re-fermentation of wines. With respect to total SO*_2_*, the highest content was observed in CS12-HOTR wine at 3 months and in TE12-HOTR wine at 6 months.

### 3.2. Evolution of Wines

**Figure 1:**
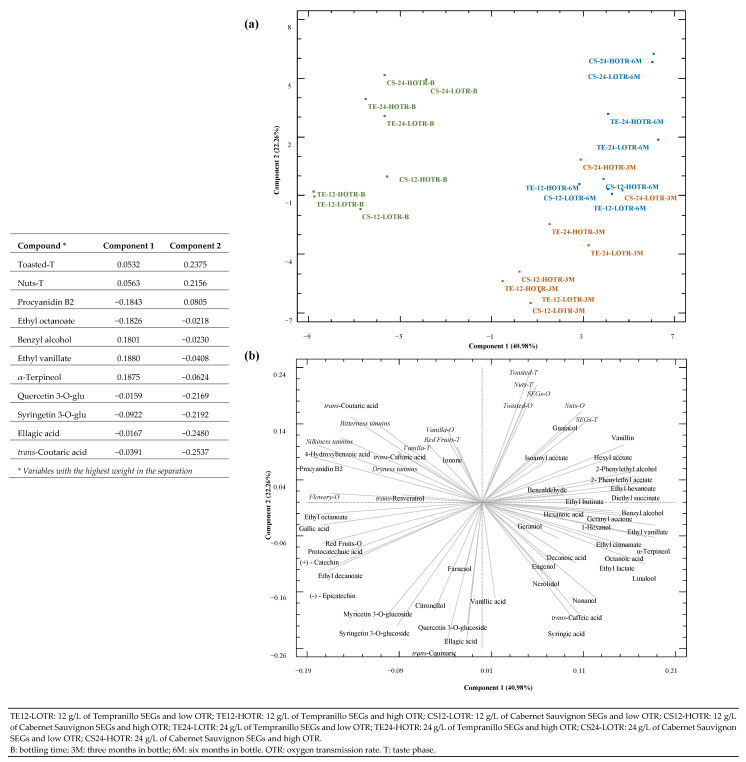
Principal component analysis (PCA) of wines using volatile and phenolic compounds and sensorial analysis descriptors at bottling time and after remaining 3 and 6 months in the bottle. (**a**) Projection in the plane of the different wines (**b**) Projection in the plane of the different variables.

To study the chemical and sensorial evolution of wines throughout the bottle ageing period, as well as the weight of the different variables in this evolution, a principal component analysis (PCA) was carried out considering the volatile and phenolic composition and sensorial descriptors of wines, and the results are summarised in Figure 1, where the first two main components were chosen to visualise the layout of the samples at the variable level. Thus, two figures were obtained: the first one (Figure 1a) showed the projection of wines on the plane factor, and the second (Figure 1b) showed the projection of the different variables on the plane as well as their weight in the separation of wines.

In Figure 1a, two principal components were constructed, which explained 63.24% of the total variance, with 40.98% for component 1 and 22.26% for component 2, respectively. The variables with the greatest weight, in terms of absolute values in component 1, were ethyl vanillate, α-terpineol, procyanidin B2, ethyl octanoate, and benzyl alcohol. This component makes it possible to differentiate wines according to bottling time evolution, locating wines at their bottling time on the negative axis, with themselves after 3 and 6 months in the bottle on the positive axis. Moreover, the sensorial descriptors associated with the use of SEGs in wines (SEGs, nuts, and toasted) were in this part of the component 1, so it could be said that the greatest evolution in the bottle, related to the effect of SEGs on the sensorial profile of wines, takes place after 6 months in the bottle. Regarding the second component, the variables with the greatest weight were toasted and nuts descriptors, at the taste phase, and quercetin-3-*O*-glucoside, syringetin 3-*O*-glucoside, ellagic acid, and *trans*-coumaric acid compounds. About this component, in the positive part of the axis, wines elaborated with the highest dose of SEGs (24 g/L) and both doses of MOX were found, both for the time of bottling, and for those aged 6 months in the bottle. Thus, wines elaborated with 12 g/L of SEGs and analysed at bottling time were placed on the negative side of this component. Also, wines after 3 months in the bottle, being those from the highest doses of SEGs (24 g/L), and both doses of MOX (LOTR and HOTR) were more dispersed than those from the 12 g/L dose. In the separation of these wines, chemical composition had more weight compared to those located on the upper side of the PCA, in which the sensorial descriptors have more weight. Therefore, considering the PCA distribution of wines, the discussion of the results was separated into two parts: first, the evolution of wines from bottling to 3 months in the bottle (lower-right quadrant of the PCA); and second, the evolution of the wines from 3 to 6 months in the bottle (upper-right quadrant of the PCA).

Regarding wines after 3 months in the bottle, the main compounds with the highest weight in the separation were studied: (a) Table 3 shows the *p*-values obtained by the contrast of compounds concentration of each wine after 3 months in the bottle with respect to itself at bottling time; and (b) Table 4 summarises the compounds concentrations as well as the variations with respect to itself at bottling time (in brackets). In this last case, two ANOVAs were carried out: (i) to know the MOX dose that most significantly resulted in the variation in the compound concentrations under the same SEGs dose, statistical analysis was carried out comparing the variation observed for each MOX dose with respect to each wine; (ii) to know the SEGs dose that resulted in the most significant variation in the compound concentrations under the same MOX dose, statistical analysis was carried out comparing the variation observed for each SEGs dose with respect to each wine.

In general, an increase in the concentration of these compounds was observed regarding the bottling time, with acids, alcohols, and esters showing the highest increase. Regarding acids, all of them increased during 3 months in the bottle, except decanoic acid with 12 g/L and HOTR for both varieties (Table 4), for which no significant differences were observed (Table 3). For all acids, Table 4 shows that wines with the same SEGs dose (12 or 24 g/L) showed a greater increase when combined with LOTR rather than HOTR, except for wines at the 24 g/L dose of Cabernet Sauvignon SEGs, which did not show significant differences. In addition, hexanoic and octanoic acids showed a greater increase during the 3 months of bottle time when the added dose of vine-shoots increased (more with 24 g/L than with 12 g/L), being statistically significant when a higher MOX dose was considered and more evident for Cabernet Sauvignon SEGs. However, such affect was not clearly observed for decanoic acid. Octanoic acid exceeded its olfactory perception threshold, which is 500 µg/L [11]. However, the impact of this compound on wine flavour can be pleasant or unpleasant, depending on the concentration. Studies have reported that these compounds can compromise the wine´s aroma at concentrations over 20 mg/L [27]. Therefore, despite their increase, especially in wines with low SEGs-MOX doses, the acids concentration remained below this value. The highest increase with the lowest SEGs-MOX combination observed in these compounds when Tempranillo SEGs were used could be due to several factors. One of them point out that the degradation of these compounds from oxidation reactions increases with higher MOX doses, combined with the absorption by SEGs, as noted by some authors [3]. However, this trend was not observed in the 24 g/L dose of SEGs of the Cabernet Sauvignon variety, and further studies are needed to explain the observed effect.

The group of alcohols was represented by benzyl alcohol, 1-hexanol, and nonanol (Table 3 and Table 4). In general, these compounds significantly increased in all cases during bottle storage, with 1-hexanol being the one that was least affected by the 3-months ageing in the bottle (Table 3). Thus, this last compound behaved differently based on the SEGs variety: for Tempranillo SEGs, it increased with the lowest SEGs dose, whereas for Cabernet Sauvignon SEGs, its increment was slightly higher when a 24 g/L dose of SEGs was used. So, the behaviour of this alcohol could not be associated with either the SEGs or MOX dose. Benzyl alcohol showed a greater concentration increment when the higher SEGs doses were combined with both MOX dosages (Table 3). However, only for Tempranillo SEGs and HOTR was this significative (Table 4). Nonanol trend depended on the SEGs-MOX combination. Thus, wines with 12 g/L of SEGs showed a greater increase when receiving lower MOX dosages, whereas wines with a higher dose of SEGs (24 g/L) and HOTR were higher, although these differences were only significant for the 12 g/L dose of Tempranillo SEGs (Table 4). For all alcohols, Table 4 showed that wines with the higher SEGs doses showed a greater significant increase when combined with HOTR rather than LOTR, except for wines with 24 g/L of Tempranillo SEGs, which did not show significant differences.

The second most abundant group of compounds that had higher weight in the evolution of the wines after 3 months in the bottle were esters. All of them showed a significant concentration increase from bottling time, except to C12-HOTR wines for ethyl cinnamate and ethyl lactate (Table 3). In general, ethyl cinnamate increment was greater at a higher SEGs dose, but without significant differences among both MOX dosages. However, with 12 g/L of SEGs for both varieties, statistically significant differences were observed, the LOTR wines being those that presented significantly high increment values (Table 4). The increase in ethyl cinnamte could be associated with one of its precursors, 4-hydroxybenzoic acid [28], located in the upper left of the PCA (Figure 1b), which was detected only in wines at bottling time, and not after bottle ageing. The higher increase in such ester, especially at lower SEGs-MOX doses, could be associated with the oxygen level affecting precursor consumption before ester formation, but a lower oxygen level might mitigate these reactions, favouring the esterification step. The ethyl lactate compound is associated with lactic acid production through esterification in the presence of ethanol [29]. Its increment was not related to the SEGs dose for both vine-shoots varieties studied, but a greater increase was observed in HOTR ones rather than in LOTR, being only significant for Tempranillo SEGs (Table 4). However, in the wines treated with the Cabernet Sauvignon variety, the SEGs dose showed more effect, with a greater increase, being observed when the higher dose was used (24 g/L), but without significant effect among MOX. Ethyl vanillate did not show a clear trend related to SEGs dose, but it was clearly affected by the MOX dose (Table 4), since its increment was significantly higher in LOTR wines rather than HOTR ones, independently of the SEGs variety (Table 3).

Regarding terpenes, an important group of compounds mainly responsible for the varietal aroma of wines, which convey their floral and sweet aroma [30,31], increase their content in most of wines with bottle ageing, which can be attributed to the hydrolysis of their glycosidic precursors [32]. Table 3 shows a significant increase in terpenes for all SEGs-MOX combinations, but not for geraniol, for which differences were only observed for 12 g/L of Tempranillo SEGs, and for geranyl acetone, for which only wines with 24 g/L of Cabernet Sauvignon SEGs and HOTR were not significant. In general, geranyl acetone increases after 3 months in bottle, more when a lower MOX dose was considered, being only significant for 24 g/L of Cabernet Sauvignon SEGs (Table 4). Also, for this compound and for the same MOX dosage, when a greater SEGs dose was considered, a lower increment was observed, being only significant for Cabernet Sauvignon variety (Table 4). For linalool, wines treated with LOTR presented a higher increment after 3 months in the bottle than those treated with HOTR, being such increment statistically significant except for wines treated with 24 g/L of Cabernet Sauvignon SEGs. Nerolidol increments after 3 months in the bottle similar for all wines, so no differences could be observed for either SEGs or MOX doses. α-Terpineol increment was significant at least with a *p* < 0.01 for all wines (Table 3). However, its greater increment was observed at a higher SEGs dose combined with the lower MOX dose, but no significant differences were observed between these variations for the two MOX or for the two SEGs doses (Table 4). Some authors have reported a positive effect of MOX in the increase in terpenes concentration [33], but, in this work, no clear MOX effect was observed, maybe because other factors such as SEGs dose and variety may also have influenced it.

Regarding volatile phenols, only eugenol contributed to the differentiation of wines after 3 months in the bottle. This compound, associated with spicy notes, exceeded its perception threshold (6 µg/L) [34] in all wines, but its behaviour varied depending on the SEGs variety considered. Wines treated with Tempranillo SEGs showed an increase in eugenol content compared to bottling time. However, for those treated with Cabernet Sauvignon SEGs, only a significant increase was observed when 12 g/L of SEGs and LOTR were combined (Table 3). For Tempranillo SEGs, the increment in eugenol was the highest for a dose of 24 g/L and lower MOX (LOTR), showing significant differences (Table 4). This behaviour may be because MOX negatively affects certain compounds through oxidation or other undesired reactions.

The content of the phenolic compounds *trans*-caffeic and syringic acids increased during the 3 months in bottle in all cases, but vanillic acid increment was only significantly for wines treated with the lower SEGs dose (Table 3). *trans*-Caffeic acid showed a tendency of greater increase with HOTR for the Tempranillo variety and with LOTR for the Cabernet Sauvignon one, although when the significance of the MOX effect was studied separately from the SEGs dose, there were no significant differences. However, when the increment was compared for the same MOX, significant differences were observed among SEGs doses, the wines treated with the lower SEGs dose having a higher increment (Table 4). For syringic acid, in general, the increments were higher when the HOTR was considered, but without significant differences when MOX was studied separately from the SEGs dose. For vanillic acid, although no statistically significant differences were observed when comparing the increments with respect to bottling between the two MOX doses, a trend was observed: at a lower SEGs dose and HOTR, the increment was higher for both varieties.

Regarding the evolution of wines after 6 months in the bottle, located in the upper-right quadrant of the PCA (Figure 1a), compounds with more weight in their evolution are shown in Table 5 and Table 6. So, in a similar way to the wines aged for 3 months, Table 5 shows the *p*-values obtained by the contrast of compound concentrations of each wine after 6 months in the bottle with respect to itself at 3 months in the bottle and Table 6 summarises the statistical analysis (ANOVA) that was carried out comparing the variation (in brackets) observed for each wine depending on the MOX or SEGs factors. In general, the selected compounds did not show a clear trend, as behaviours varied by SEGs variety, and only the guaiacol increased significatively in all wines (Table 5). Also, if these results are compared with those in Table 3, a lower number of significant increases were observed, which highlights a lower evolution among the wines between 3 and 6 months, compared with the evolution between bottling time and 6 months in the bottle, as indicated in the PCA (Figure 1).

Benzaldehyde concentration significantly decreased from 3 to 6 months in the bottle, but not clear trend was observed regarding SEGs and MOX doses (Table 6). The most abundant compound in these wines was 2-phenylethyl alcohol, which only significantly increased when the lowest SEGs dose of Cabernet Sauvignon was used (Table 5), but no significant differences were observed when the MOX effect was studied separately from the SEGs dose (Table 6).

The esters were the most affected group of compounds, with diethyl succinate being the most abundant (Table 5). Its increment was significant in wines treated with the SEGs from Cabernet Sauvignon variety (Table 6). Diethyl succinate showed a greater increase at a higher SEGs dose as well as at a higher MOX dose, except for wines treated with 12 g/L of Tempranillo variety, where a decrease in this compound was observed due to a higher MOX dose. So, it could be suggested that higher MOX dosages are responsible for increasing or maintaining the diethyl succinate content. Also, for Tempranillo SEGs and for both MOX doses, higher significant increments were observed among SEGs doses, being higher for wines treated with 24 g/L. Some studies suggest that vine-shoots are a source for succinic acid production [35], so this behaviour could be due to the higher succinic acid levels from Cabernet Sauvignon SEGs, and its esterification in the presence of ethanol being favoured by micro-oxygenation. Regarding the ethyl esters, butyrate and hexanoate, no clear trend with SEGs-MOX treatments was observed (Table 5 and Table 6). The ethyl butyrate concentration was affected by MOX at the highest oxygen dose in the case of Cabernet Sauvignon, but only increased significantly for the highest SEGs dose. For Tempranillo SEGs, the behaviour was different for 24 g/L. As for the previous, the ethyl hexanoate behaved differently between varieties: for Tempranillo SEGs, the highest increase was observed with 24 g/L and LOTR, whereas for Cabernet Sauvignon the highest increase was observed with the high MOX (HOTR) (Table 6). This compound forms from hexanoic acid esterification, so the fact that it had the highest hexanoic acid content could explain the higher increase in ethyl hexanoate in these wines. As for the acetates, hexyl, isoamyl, and 2-phenylethyl, they did not show a clear trend either, although in all cases these compounds increased when the higher-dose SEGs-MOX combination was used. This highlighted the isoamyl acetate, associated with the banana aroma, whose values were higher than its odour threshold (30 µg/L) in all wines [36].

The most significant volatile phenols differentiating wines after 6 months in the bottle were guaiacol and vanillin, mainly derived from the lignin degradation of vine-shoots [37,38]. Guaiacol increased significantly in all wines at 6 months compared to 3 months, with the highest increase (169.50%) in Cabernet Sauvignon with a higher SEGs-MOX dose, and a lower increase (97.80%) in Tempranillo with a lower dose. The increase in Tempranillo SEGs was greater at lower SEGs and MOX doses, contrary to the Cabernet Sauvignon variety, where a higher increase was observed at higher SEGs and MOX doses (Table 6). For vanillin, the highest increment was observed with the higher SEGs and MOX doses, being significantly different for all wines except for those treated with 24 g/L of Tempranillo SEGs.

**Figure 2:**
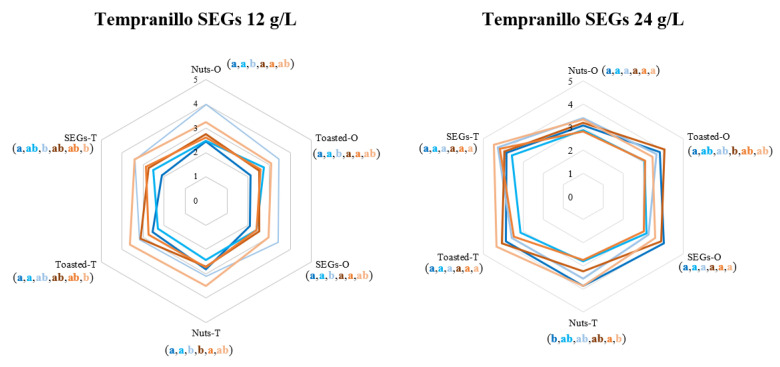

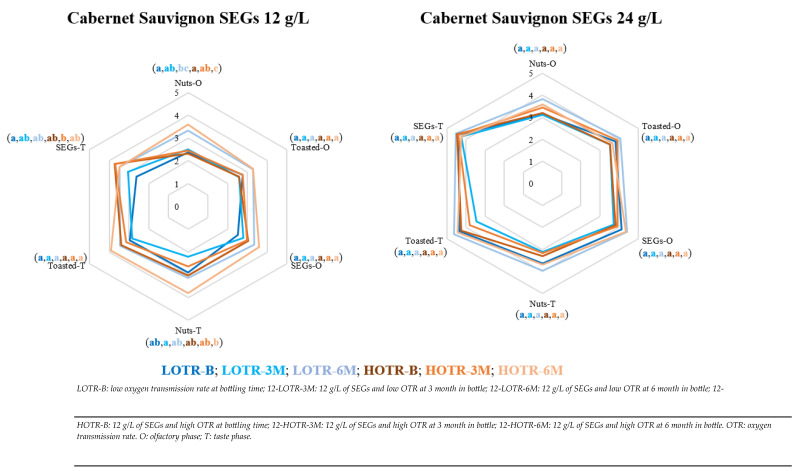
Evolution of the olfactory and tasted sensorial descriptors of SEGs-MOX-treated wines at bottling time and after remaining 3 and 6 months in the bottle.

In the sensorial analysis of wines, the olfactory and taste descriptors with the greatest importance in the evolution of wines during the 6 months in the bottle were nuts, toasted, and SEGs notes (Figure 1b). Therefore, Figure 2 shows, in spider charts, how these descriptors evolve from the moment of bottling until 6 months in the bottle. To determine differences, one-way ANOVA was performed for each descriptor, comparing the two MOX doses at each of the three sampling moments (bottling, 3 and 6 months in the bottle) for each SEGs variety (Tempranillo and Cabernet Sauvignon) and SEGs dose (12 and 24 g/L). In general, it is important to note that, when the highest dosage of SEGs (24 g/L) was used, a larger and more balanced area was observed in the diagrams from the beginning of the tastings, which indicates a greater roundness of the wine and, at the same time, a greater intensity of the descriptors.

The SEGs descriptor at taste phase, linked to a “sweet woody” note [7], was influenced by oxygen dosage and bottle ageing when a 12 g/L dose of SEGs was used. For Tempranillo SEGs, it was more intense with the highest dose after 6 months, for both MOX doses. For Cabernet Sauvignon SEGs, the highest MOX dose resulted in greater intensity for the panellists, with little difference over bottle time.

On the other hand, the toasted descriptor at the taste phase only showed significant differences when the Tempranillo variety was used at the lowest SEGs dose (12 g/L). Thus, tasters perceived significantly more intense toasted notes after 6 months in the bottle in wine with the highest MOX dose, which could be associated with a higher release of aromas from the vine-shoots due to the higher amount of oxygen.

Nuts attribute was associated by tasters with hazelnut and almond shells and differences were only observed between the wines when the lowest dosage of SEGs was used. Thus, for the Tempranillo variety, the greatest intensity was perceived at 6 months in the bottle and the lowest MOX dose, while for the Cabernet Sauvignon variety, the greatest intensity was seen at the highest MOX dose.

## 4. Conclusions

The effect of SEGs from different varieties combined with MOX treatments was studied during bottled ageing (3 and 6 months) attending to phenolic and volatile composition as well as the sensorial profile of Tempranillo wines. The results did not show a clear impact of MOX treatments on the chemical profile of these wines, so, this effect seems to be highly dependent on the vine-shoots variety and the specific SEGs-MOX combination used. After 3 months in the bottle, the chemical profile was more significantly modified with a general increase in most of the analysed compounds, both phenolic and volatile, compared to bottling time. After 6 months in the bottle, the greatest effect was perceived in the sensorial profile, with better integration of the different descriptors analysed and greater roundness in the wines. Differences were only found in the SEGs, nut, and toasted notes at the taste phase, which were specific descriptors associated with the use of vine-shoots as oenological additives.

## Figures and Tables

**Table 1:** Wine treatments according to the SEG-MOX combinations.

SEGs Variety	SEGs Dose	MOX Dose	Code
Tempranillo	Low	Low	TE12-LOTR
Tempranillo	Low	High	TE12-HOTR
Tempranillo	High	Low	TE24-LOTR
Tempranillo	High	High	TE24-HOTR
Cabernet Sauvignon	Low	Low	CS12-LOTR
Cabernet Sauvignon	Low	High	CS12-HOTR
Cabernet Sauvignon	High	Low	CS24-LOTR
Cabernet Sauvignon	High	High	CS24-HOTR

**Table 2:** Oenological parameters of Tempranillo wines treated with SEGs and MOX after remaining in the bottle for 3 and 6 months.

***	Bottle Ageing Time (Months)	TE12-LOTR	TE12-HOTR	TE24-LOTR	TE24-HOTR	CS12-LOTR	CS12-HOTR	CS24-LOTR	CS24-HOTR
**AC (% *v*/*v*)**	3	**14.3 ± 0.0 d**	14.3 ± 0.0 d	**14.2 ± 0.0 bc**	14.2 ± 0.0 a	14.2 ± 0.1 bc	14.2 ± 0.0 c	14.2 ± 0.0 b	14.2 ± 0.0 ab
	6	**14.3 ± 0.0 d**	14.3 ± 0.0 cd	14.3 ± 0.0 abc	14.3 ± 0.0 ab	14.3 ± 0.1 bcd	14.3 ± 0.0 ab	14.3 ± 0.0 a	14.3 ± 0.0 abc
**TA (g/L)**	3	4.0 ± 0.0 ab	4.0 ± 0.0 bc	4.0 ± 0.1 bc	4.0 ± 0.0 ab	4.0 ± 0.0 ab	**4.1 ± 0.3 c**	3.9 ± 0.0 ab	3.9 ± 0.0 a
	6	**3.9 ± 0.0 c**	4.0 ± 0.1 bc	4.0 ± 0.1 bc	3.9 ± 0.0 abc	4.0 ± 0.1 abc	**4.1 ± 0.4 c**	3.8 ± 0.0 a	3.8 ± 0.0 ab
**pH**	3	4.2 ± 0.0 ab	4.2 ± 0.0 a	4.2 ± 0.0 bc	**4.2 ± 0.0 c**	4.2 ± 0.0 ab	4.2 ± 0.0 a	4.2 ± 0.0 a	**4.3 ± 0.0 c**
	6	4.1 ± 0.0 a	4.1 ± 0.0 a	4.1 ± 0.0 a	4.1 ± 0.0 a	4.1 ± 0.0 a	4.1 ± 0.1 a	4.1 ± 0.0 a	4.1 ± 0.0 a
**VA (g/L)**	3	0.8 ± 0.0 a	0.8 ± 0.0 abcd	0.8 ± 0.1 cd	0.8 ± 0.0 bcd	0.7 ± 0.0 a	**0.9 ± 0.1 d**	0.8 ± 0.0 ab	0.8 ± 0.0 abc
	6	0.8 ± 0.0 a	0.8 ± 0.0 ab	0.8 ± 0.0 abc	**0.9 ± 0.1 c**	0.8 ± 0.0 ab	**0.9 ± 0.1 c**	0.8 ± 0.0 bc	**0.9 ± 0.0 c**
**Reducing sugars (g/L)**	3	1.8 ± 0.0 a	1.8 ± 0.1 a	2.4 ± 0.2 b	**2.4 ± 0.4 b**	1.9 ± 0.2 a	1.7 ± 0.2 a	**2.4 ± 0.2 b**	**2.4 ± 0.2 b**
	6	1.9 ± 0.0 a	1.8 ± 0.1 a	2.3 ± 0.1 b	**2.5 ± 0.3 b**	1.8 ± 0.1 a	1.8 ± 0.5 a	**2.5 ± 0.1 b**	**2.5 ± 0.3 b**
**Total SO_2_ (mg/L)**	3	62.0 ± 3.6 c	63.3 ± 1.4 cd	52.0 ± 1.8 a	58.0 ± 5.0 b	66.0 ± 0.9 de	**68.3 ± 1.4 e**	57.7 ± 3.6 b	56.7 ± 1.9 b
	6	63.0 ± 0.9 bc	**64.0 ± 1.8 c**	57.3 ± 1.4 bc	60.3 ± 2.1 bc	61.3 ± 2.2 bc	59.7 ± 2.2 bc	56.0 ± 1.8 b	47.0 ± 18.6 a

SEGs: Shoot from vines—Enological—Granule; OTR: oxygen transmission rate. TE12-LOTR: 12 g/L of Tempranillo SEGs and low OTR; TE12-HOTR: 12 g/L of Tempranillo SEGs and high OTR; CS12-LOTR: 12 g/L of Cabernet Sauvignon SEGs and low OTR; CS12-HOTR: 12 g/L of Cabernet Sauvignon SEGs and high OTR; TE24-LOTR: 24 g/L of Tempranillo SEGs and low OTR; TE24-HOTR: 24 g/L of Tempranillo SEGs and high OTR; CS24-LOTR: 24 g/L of Cabernet Sauvignon SEGs and low OTR; CS24-HOTR: 24 g/L of Cabernet Sauvignon SEGs and high OTR. AC (% *v*/*v*): alcohol concentration; TA: total acidity, expressed as g/L tartaric acid; VA: volatile acidity, expressed as g/L acetic acid. The mean values (n = 3) are shown with their standard deviation. For each parameter, different letters indicate significant differences between treated wines according to Fisher´s LSD test (*p* value < 0.05) and the highest values have been highlighted in bold. *** The bottling data are reported in the article by Cebrián-Tarancón et al. [18].

**Table 3:** *p*-values obtained by comparing the concentration of each compound (µg/L or mg/L) for each treated wine after remaining in the bottle for 3 months.

	TE12-3m		TE24-3m		CS12-3m		CS24-3m	
	LOTR	HOTR	LOTR	HOTR	LOTR	HOTR	LOTR	HOTR
**Volatile compounds (µg/L)**								
**Acids**								
Decanoic acid	***		**	**	***		**	*
Hexanoic acid	***	***	***	**			**	**
Octanoic acid	***	**	**	**	**		***	**
**Alcohols**								
Benzyl alcohol	***	***	***	***	***	***	***	***
1-Hexanol	*	*					**	**
Nonanol	***	***	***	***	***	***	***	***
**Esters**								
Ethyl cinnamate	*	***	***	***	***		***	***
Ethyl lactate	*	**	**	***	*		**	**
Ethyl vanillate	**	**	***	***	***	***	***	***
**Terpenes**								
Geraniol	**	**						
Geranyl acetone	**	***	***	***	***	***	*	
Linalool	***	***	***	*	***	***	***	***
Nerolidol	***	***	***	***	***	**	***	***
α-Terpineol	**	***	***	***	***	***	***	***
**Volatile phenols**								
Eugenol	***	***	***	***	**			
**Phenolic compounds (mg/L)**								
***trans***-Caffeic acid	***	***	***	***	***	***	***	***
Syringic acid	***	***	**	**	***	***	***	***
Vanillic acid	**	**			*	*		

SEGs: Shoot from vines—Enological—Granule; OTR: oxygen transmission rate. TE12-LOTR: 12 g/L of Tempranillo SEGs and low OTR; TE12-HOTR: 12 g/L of Tempranillo SEGs and high OTR; CS12-LOTR: 12 g/L of Cabernet Sauvignon SEGs and low OTR; CS12-HOTR: 12 g/L of Cabernet Sauvignon SEGs and high OTR; TE24-LOTR: 24 g/L of Tempranillo SEGs and low OTR; TE24-HOTR: 24 g/L of Tempranillo SEGs and high OTR; CS24-LOTR: 24 g/L of Cabernet Sauvignon SEGs and low OTR; CS24-HOTR: 24 g/L of Cabernet Sauvignon SEGs and high OTR. 3m: 3 months. For each compound and each treated wine, significant differences between bottling time and 3 months of bottle are considered according to Fisher´s LSD test (* *p* value < 0.05; ** *p* value < 0.01; *** *p* value < 0.001).

**Table 4:** Volatile (µg/L) and phenolic (mg/L) compounds with more weight in the differentiation of wines after remaining in the bottle for 3 months.

	TE12-3m		TE24-3m		TE SEGs Dose		CS12-3m		CS24-3m		CS SEGs Dose	
	LOTR	HOTR	LOTR	HOTR	LOTR	HOTR	LOTR	HOTR	LOTR	HOTR	LOTR	HOTR
**Volatile compounds (µg/L)**												
**Acids**												
Decanoic acid	**67.3 ± 4.0 (19.1, b)**	48.9 ± 5.9 (−0.1, a)	**63.4 ± 6.8 (12.9, b)**	48.9 ± 4.9 (8.2, a)	A, A	A, B	**77.3 ± 9.1 (18.4, b)**	51.3 ± 3.2 (−1.7, a)	52.9 ± 6.8 (8.4, a)	45.3 ± 8.2 (8.5, a)	B, A	A, B
Hexanoic acid	**1798.3 ± 65.3 (538.6, b)**	1597.9 ± 65.3 (167.1, a)	**2204.6 ± 196.6 (672.2, b)**	1744.8 ± 197.4 (264.6, a)	A, A	A, A	1890.0 ± 209.1 (163.6, -)	1621.8 ± 96.8 (13.2, -)	2225.8 ± 275.0 (332.6, a)	1859.8 ± 238.5 (293.0, a)	A, B	A, B
Octanoic acid	**668.8 ± 7.0 (188.5, b)**	593.6 ± 53.3 (71.9, a)	**674.2 ± 105.6 (145.1, b)**	616.9 ± 112.4 (131.0, a)	A, A	A, A	**686.6 ± 55.2 (116.9, b)**	584.9 ± 41.9 (19.0, a)	637.5 ± 67.2 (118.2, a)	580.9 ± 95.2 (127.6, a)	A, A	A, B
**Alcohols**												
Benzyl alcohol	1592.3 ± 71.1 (759.2, a)	1563.6 ± 192.8 (651.6, a)	**2443.7 ± 375.2 (1430.2, b)**	2147.6 ± 300.7 (1152.7, a)	A, B	A, B	1732.2 ± 185.5 (761.0, a)	1552.9 ± 74.6 (483.5, a)	2079.1 ± 315.5 (996.9, a)	1910.0 ± 231.3 (942.2, a)	A, A	A, B
1-Hexanol	1950.2 ± 75.2 (151.9, a)	1960.5 ± 191.4 (201.6, a)	1840.6 ± 140.4 (−0.1, -)	2031.0 ± 200.4 (85.8, -)	B, A	A, A	1915.0 ± 63.4 (27.5, -)	1966.6 ± 105.8 (8.2, -)	2083.8 ± 178.9 (260.9, a)	2055.4 ± 250.6 (260.4, a)	A, B	A, B
Nonanol	**3.8 ± 0.0 (3.2, b)**	3.1 ± 0.4 (1.1, a)	3.6 ± 0.9 (1.5, a)	3.3 ± 0.7 (2.0, a)	B, A	A, B	4.7 ± 0.5 (2.5, a)	3.3 ± 0.4 (0.8, a)	4.0 ± 0.5 (2.3, a)	3.9 ± 0.6 (2.4, a)	A, A	A, B
**Esters**												
Ethyl cinnamate	**0.5 ± 0.0 (0.3, b)**	0.4 ± 0.1 (0.2, a)	0.5 ± 0.1 (0.3, a)	0.5 ± 0.1 (0.3, a)	A, A	A, B	**0.4 ± 0.1 (0.2, b)**	0.4 ± 0.2 (0.2, a)	0.5 ± 0.1 (0.2, a)	0.4 ± 0.1 (0.2, a)	A, A	A, A
Ethyl lactate	5980.4 ± 169.9 (918.0, a)	**5650.7 ± 696.7 (1298.3, b)**	5574.5 ± 353.9 (483.4, a)	**5925.1 ± 613.3 (1352.1, b)**	A, A	A, A	6038.2 ± 442.3 (506.5, a)	5420.4 ± 211.3 (625.7, a)	6025.7 ± 534.5 (839.4, a)	5380.8 ± 625.7 (722.2, a)	A, A	A, A
Ethyl vanillate	**215.6 ± 16.5 (79.8, b)**	203.1 ± 29.2 (44.4, a)	**222.4 ± 30.1 (69.7, b)**	187.8 ± 15.4 (34.3, a)	A, A	A, A	**224.6 ± 16.4 (67.8, b)**	201.9 ± 17.8 (31.9, a)	**230.9 ± 37.9 (72.9, b)**	203.1 ± 27.3 (50.6, a)	A, A	A, A
**Terpenes**												
Geraniol	9.4 ± 0.2 (1.4, a)	8.9 ± 0.8 (1.2, a)	9.5 ± 0.7 (0.9, -)	9.4 ± 1.4 (0.6, -)	A, A	A, A	9.2 ± 0.9 (1.0, -)	10.0 ± 1.4 (1.0, -)	10.4 ± 0.9 (0.9, -)	10.0 ± 1.1 (0.4, -)	A, A	A, A
Geranyl acetone	1.7 ± 0.1 (0.3, a)	1.6 ± 0.1 (0.3, a)	1.8 ± 0.1 (0.3, a)	1.7 ± 0.1 (0.2, a)	A, A	A, A	1.8 ± 0.1 (0.3, a)	1.7 ± 0.1 (0.3, a)	**1.9 ± 0.1 (0.2, b)**	1.7 ± 0.1 (-0.1, a)	B, A	B, A
Linalool	**5.4 ± 0.4 (1.3, b)**	5.3 ± 0.3 (1.2, a)	**5.7 ± 0.7 (1.6, b)**	5.1 ± 0.8 (0.8, a)	A, A	A, A	**5.4 ± 0.4 (1.3, b)**	5.5 ± 0.8 (1.2, a)	5.5 ± 0.3 (1.4, a)	5.3 ± 0.5 (1.3, a)	A, A	A, A
Nerolidol	1.7 ± 0.1 (0.3, a)	1.7 ± 0.1 (0.3, a)	2.2 ± 0.6 (0.4, a)	1.7 ± 0.1 (0.3, a)	A, A	A, A	1.7 ± 0.1 (0.2, a)	1.7 ± 0.2 (0.2, a)	1.6 ± 0.1 (0.3, a)	1.6 ± 0.1 (0.2, a)	A, A	A, A
α-Terpineol	9.7 ± 0.5 (3.0, a)	9.7 ± 0.8 (2.8, a)	11.0 ± 1.1 (3.8, a)	10.0 ± 0.9 (2.8, a)	A, B	A, A	9.9 ± 0.9 (2.9, a)	**10.2 ± 1.4 (3.1, b)**	10.4 ± 0.8 (3.4, a)	10.3 ± 0.8 (3.4, a)	A, A	A, A
**Volatile phenols**												
Eugenol	**10.7 ± 0.4 (2.8, b)**	10.0 ± 0.6 (1.9, a)	**12.4 ± 1.2 (3.6, b)**	11.9 ± 1.7 (3.3, a)	A, A	A, B	9.2 ± 0.9 (1.3, a)	8.5 ± 1.0 (0.7, a)	8.6 ± 1.0 (0.6, a)	8.3 ± 1.1 (0.7, a)	A, A	A, A
**Phenolic compounds (mg/L)**												
**Phenolic acids**												
***trans***-Caffeic acid	11.5 ± 0.1 (6.1, a)	12.4 ± 0.8 (6.8, a)	10.7 ± 1.0 (5.3, a)	11.4 ± 1.1 (6.1, a)	B, A	A, A	11.3 ± 1.4 (5.8, a)	11.7 ± 1.6 (6.4, a)	9.5 ± 1.2 (4.4, a)	10.6 ± 1.2 (5.7, a)	B, A	A, A
Syringic acid	7.1 ± 0.2 (0.2, a)	7.2 ± 0.3 (0.3, a)	7.0 ± 0.2 (0.2, a)	7.0 ± 0.3 (0.3, a)	B, A	A, A	7.4 ± 0.3 (0.9, a)	7.4 ± 0.3 (1.0, a)	7.2 ± 0.2 (1.0, a)	7.0 ± 0.3 (0.8, a)	A, A	A, A
Vanillic acid	4.0 ± 0.1 (0.2, a)	3.9 ± 0.1 (0.3, a)	3.9 ± 0.2 (0.2, a)	3.9 ± 0.1 (0.0, a)	A, A	B, A	3.9 ± 0.1 (0.2, a)	3.9 ± 0.1 (0.2, a)	3.9 ± 0.1 (0.1, a)	3.9 ± 0.2 (0.1, a)	B, A	A, A

SEGs: Shoot from vines—Enological—Granule; OTR: oxygen transmission rate. TE12-LOTR: 12 g/L of Tempranillo SEGs and low OTR; TE12-HOTR: 12 g/L of Tempranillo SEGs and high OTR; CS12-LOTR: 12 g/L of Cabernet Sauvignon SEGs and low OTR; CS12-HOTR: 12 g/L of Cabernet Sauvignon SEGs and high OTR; TE24-LOTR: 24 g/L of Tempranillo SEGs and low OTR; TE24-HOTR: 24 g/L of Tempranillo SEGs and high OTR; CS24-LOTR: 24 g/L of Cabernet Sauvignon SEGs and low OTR; CS24-HOTR: 24 g/L of Cabernet Sauvignon SEGs and high OTR.. In brackets, the variations (µg/L or mg/L) were calculated with respect to wine at bottling time as content at 3 months minus the initial content, where negative value means loss of content after 3 months in the bottle and positive value means increase in content after 3 months in the bottle. The mean values (n = 3) are shown with their standard deviation. For each compound, SEGs variety, and SEGs doses, small letters indicate significant differences among the variations at 3 months in the bottle with respect to itself at bottling time between the two OTR doses. Capital letters indicate significant differences among the variations of 3 months in the bottle with respect to itself at bottling time between the two SEGs doses at same OTR doses according to Fisher´s LSD test (*p* value < 0.05) and the higher value was highlighted in bold. “-” means that statistics have not been carried out because no significant differences are observed in Table 3.

**Table 5:** *p*-value obtained by comparing the concentration of each compound (µg/L) for each treated wine after remaining in the bottle for 6 months.

	TE12-6m		TE24-6m		CS12-6m		CS24-6m	
	LOTR	HOTR	LOTR	HOTR	LOTR	HOTR	LOTR	HOTR
**Aldehydes**								
Benzaldehyde	***	**	***	***	*	***	*	
**Alcohols**								
2-Phenylethyl alcohol					***	***		
**Esters**								
Diethyl succinate	***			*	**	***	**	***
Ethyl butyrate					*			
Ethyl hexanoate			**	**	**			*
Hexyl acetate	*	*			***	**		***
Isoamyl acetate			*	***	***	**		*
2-Phenylethyl acetate	**	***		**	*	***		***
**Volatile phenols**								
Guaiacol	***	**	***	**	***	***	**	***
Vanillin		***		*	***	***	*	***

SEGs: Shoot from vines—Enological—Granule; OTR: oxygen transmission rate. TE12-LOTR: 12 g/L of Tempranillo SEGs and low OTR; TE12-HOTR: 12 g/L of Tempranillo SEGs and high OTR; CS12-LOTR: 12 g/L of Cabernet Sauvignon SEGs and low OTR; CS12-HOTR: 12 g/L of Cabernet Sauvignon SEGs and high OTR; TE24-LOTR: 24 g/L of Tempranillo SEGs and low OTR; TE24-HOTR: 24 g/L of Tempranillo SEGs and high OTR; CS24-LOTR: 24 g/L of Cabernet Sauvignon SEGs and low OTR; CS24-HOTR: 24 g/L of Cabernet Sauvignon SEGs and high OTR. 6m: 6 months. For each compound and each treated wine significant differences between 3 and 6 months of bottle are considered according to Fisher´s LSD test (* *p* value < 0.05; ** *p* value < 0.01; *** *p* value < 0.001).

**Table 6:** Volatile compounds (µg/L) with more weight in the differentiation of wines after remaining 6 months in the bottle.

	TE12-6m		TE24-6m		TE-SEGs Dose		CS12-6m		CS24-6m		CS-SEGs Dose	
	LOTR	HOTR	LOTR	HOTR	LOTR	HOTR	LOTR	HOTR	LOTR	HOTR	LOTR	HOTR
**Aldehydes**												
Benzaldehyde	4.3 ± 0.3 (−2.3, a)	**4.2 ± 0.5 (−1.2, b)**	**5.4 ± 0.7 (−2.2, b)**	4.8 ± 0.6 (−2.6, a)	A, A	A, B	**7.1 ± 0.5 (−0.8, b)**	4.9 ± 0.6 (−2.4, a)	**11.8 ± 1.6 (2.8, b)**	8.2 ± 1.0 (−0.1, a)	A, B	B, A
**Alcohols**												
2-Phenylethyl alcohol	8437.0 ± 333.6 (289.8, a)	8577.7 ± 417.7 (607.1, a)	9267.2 ± 1210.9 (276.7, a)	8394.8 ± 449.9 (52.5, a)	A, A	A, A	8915.9 ± 370.2 (1251.6, a)	8885.5 ± 592.5 (1599.9, a)	8967.5 ± 723.7 (440.0, a)	9555.7 ± 1369.9 (1253.5, a)	A, A	A, A
**Esters**												
Diethyl succinate	2617.5 ± 141.5 (−553.5, a)	**2874.2 ± 286.0 (−69.1, b)**	3370.1 ± 269.9 (71.5, a)	**3370.9 ± 255.9 (382.6, b)**	A, B	A, B	3512.4 ± 151.6 (413.0, a)	**3746.6 ± 322.1 (823.6, b)**	4201.2 ± 107.1 (561.5, a)	**4559.7 ± 522.1 (1204.3, b)**	A, A	A, A
Ethyl butyrate	143.7 ± 14.6 (−13.3, a)	**136.7 ± 9.0 (−0.0, b)**	**149.6 ± 16.0 (8.1, b)**	128.8 ± 11.2 (−2.3, a)	A, A	A, A	148.0 ± 10.9 (17.6, a)	142.0 ± 27.7 (21.3, a)	145.3 ± 24.3 (−15.0, a)	**148.7 ± 20.6 (11.3, b)**	B, A	A, A
Ethyl hexanoate	251.3 ± 12.1 (7.0, a)	233.5 ± 14.4 (18.0, a)	**271.8 ± 37.0 (61.4, b)**	239.8 ± 12.5 (26.0, a)	A, B	A, A	253.9 ± 9.2 (23.9, b)	235.7 ± 0.2 (8.0, a)	280.5 ± 18.4 (−1.2, a)	**272.7 ± 38.9 (38.2, b)**	A, A	A, B
Hexyl acetate	1.2 ± 0.1 (0.2, a)	1.1 ± 0.1 (0.1, a)	1.4 ± 0.1 (0.2, a)	1.2 ± 0.1 (0.1, a)	A, A	A, A	1.2 ± 0.1 (0.2, a)	1.4 ± 0.1 (0.2, a)	1.3 ± 0.2 (−0.0, a)	**1.4 ± 0.1 (0.3, b)**	B, A	A, A
Isoamyl acetate	235.5 ± 28.7 (−15.5, a)	204.5 ± 30.0 (3.4, a)	286.0 ± 37.0 (39.8, a)	**224.7 ± 28.9 (50.5, b)**	A, B	A, B	**259.5 ± 19.6 (54.0, b)**	237.9 ± 17.7 (32.9, a)	262.9 ± 37.4 (−16.2, a)	**244.1 ± 29.3 (32.6, b)**	B, A	A, A
2-Phenylethyl acetate	12.8 ± 0.6 (2.0, a)	10.9 ± 1.0 (2.4, a)	14.5 ± 1.3 (0.7, a)	**10.8 ± 0.7 (2.1, b)**	A, A	A, A	12.2 ± 1.4 (2.3, a)	12.5 ± 1.2 (3.3, a)	13.1 ± 1.0 (1.1, a)	**13.4 ± 1.3 (2.9, b)**	A, A	A, A
**Volatile phenols**												
Guaiacol	**13.4 ± 4.7 (6.6, b)**	10.5 ± 2.5 (4.3, a)	**10.6 ± 1.2 (3.0, b)**	9.4 ± 1.4 (2.5, a)	B, A	A, A	10.1 ± 0.9 (2.1, a)	10.6 ± 0.9 (4.2, a)	10.8 ± 2.1 (2.4, a)	**25.4 ± 4.7 (16.0, b)**	A, A	A, B
Vanillin	148.9 ± 19.8 (21.8, a)	**132.9 ± 21.3 (38.0, b)**	162.5 ± 25.9 (24.2, a)	155.1 ± 23.4 (29.3, a)	A, A	A, A	128.5 ± 16.7 (33.2, a)	**123.1 ± 14.9 (46.3, b)**	147.6 ± 11.2 (18.6, a)	**169.9 ± 24.9 (53.0, b)**	A, A	A, A

SEGs: Shoot from vines—Enological—Granule; OTR: oxygen transmission rate. TE12-LOTR: 12 g/L of Tempranillo SEGs and low OTR; TE12-HOTR: 12 g/L of Tempranillo SEGs and high OTR; CS12-LOTR: 12 g/L of Cabernet Sauvignon SEGs and low OTR; CS12-HOTR: 12 g/L of Cabernet Sauvignon SEGs and high OTR; TE24-LOTR: 24 g/L of Tempranillo SEGs and low OTR; TE24-HOTR: 24 g/L of Tempranillo SEGs and high OTR; CS24-LOTR: 24 g/L of Cabernet Sauvignon SEGs and low OTR; CS24-HOTR: 24 g/L of Cabernet Sauvignon SEGs and high OTR. In brackets, the variations (µg/L or mg/L) were calculated with respect to wine at bottling time as: content at 6 months—initial content, where negative value means loss of content after 6 months in the bottle and positive value means increase in content after 6 months of bottle permanence. The mean values (n = 3) are shown with their standard deviation. For each compound, SEGs variety and SEGs doses, small letters indicate significant differences among the variations at 6 months in the bottle with respect to itself at bottling time between the two OTR doses. Capital letters indicate significant differences among the variations at 6 months in the bottle with respect to itself at bottling time between the two SEGs doses with the same each OTR doses according to Fisher´s LSD test (*p* value < 0.05) and the highest values have been highlighted in bold.

## Data Availability

Data are contained within the article.
